# Induction of oxidative DNA damage, cell cycle arrest and p53 mediated apoptosis by calcium titanate nanoparticles in MCF-7 breast cancer cells

**DOI:** 10.1186/s12935-022-02780-y

**Published:** 2022-11-15

**Authors:** Hanan R. H. Mohamed, Maria M. H. Ibrahim, Ayman Diab

**Affiliations:** 1 Zoology Department Faculty of Science, Cairo University, Giza, Egypt; 2 Faculty of Biotechnology, October University for Modern Sciences and Arts, Giza, Egypt

**Keywords:** Calcium titanate nanoparticles, Cytotoxicity, Genotoxicity, Apoptosis, Cell cycle arrest

## Abstract

**Background:**

The distinctive properties and high activity of calcium titanate nanoparticles (CaTiO_3_-NPs) increase their use in many products. However, the cytotoxic and genotoxic effects of CaTiO_3_-NPs in human cancer cell lines have not been well studied. Therefore, this study was conducted to explore CaTiO_3_-NPs induced cytotoxicity, genomic instability and apoptosis in human breast cancer (MCF-7) cells.

**Methods:**

Sulforhodamine B (SRB) and the alkaline comet assays were done to study cell viability and DNA damage induction, respectively. Apoptosis induction and cell cycle distribution were analyzed using flow cytometry. The level of intracellular reactive oxygen species (ROS) was studied, and the expression levels of p53, Bax and Bcl2 genes were also measured.

**Results:**

The results of the Sulforhodamine B (SRB) cytotoxicity assay showed that viability of MCF-7 cells was not affected by CaTiO_3_-NPs treatment for 24 h, however, exposure to CaTiO_3_-NPs for 72 h caused concentrations dependent death of MCF-7 cells. Treatment with CaTiO_3_-NPs for 72 h caused marked increases in intracellular ROS level and induced DNA damage. Treatment of MCF-7 cells with CaTiO_3_-NPs also caused MCF-7 cell cycle arrest at the G0 and S phases and s triggered apoptosis of MCF-7 cells by causing simultaneous increases in the expression levels of apoptotic p53 and Bax genes and a decrease in the expression level of anti-apoptotic Bcl2 gene.

**Conclusion:**

Collectively, it was concluded that CaTiO_3_-NPs cause time- and concentration-dependent cytotoxic effects in human MCF-7 cells through induction of ROS generation, genomic instability and apoptosis. Thus it is recommended that further in vitro and in vivo studies are therefore recommended to understand the cytotoxic and biological effects of CaTiO_3_-NPs.

## Introduction

Titanium compounds such as titanium alloys, titanates and titanium dioxides are widely used in various biological applications. For example, the superior physical and biological properties of titanium-oxygen alloys make them successfully used in dental implant and orthopedic hip replacement. For enhancing the bonding bioactivity of titanium alloys alkaline or heat treatments are applied to form a calcium-TiO_2_ layer on the alloys surfaces, the calcium in this layer is fixed on the surface of the alloy and is thus not labile [1].

Calcium titanate (CaTiO_3_), an inorganic compound, is a diamagnetic solid known also as perovskite. Recent discovering of the ability of CaTiO_3_ to bind and release calcium ions increases the possibility of using CaTiO_3_ to deliver calcium ions in various medical applications [2, 3]. However, CaTiO_3_ induced-toxicity has been poorly studied. Monosodium calcium titanate has been found to cytotoxic to monocytes and fibroblasts [4, 5]. Moreover, monosodium titanate alone or with metal ions has been shown to inhibit cellular metabolism in different cell types [6–8].

Nanoparticles are of great scientific interest as they are effectively a bridge between bulk materials and atomic or molecular structures. A bulk material should have constant physical properties regardless of its size, but at the nanoscale this is often not the case [9]. Size-dependent physical and chemical properties are observed, e.g. surface area/mass ratio increased, thereby greatly enhancing chemical/catalytic reactivity (amongst other properties) compared to normal-sized particles of the same substance [10].

Nowadays there is increasing ongoing interest on using nanoparticles instead of bulk materials because nano-sized materials have variable size-dependent physical and chemical properties rather than the constant size independent physical properties of bulk materials. For example increased surface area/mass ratio in nanoparticles, resulting in significantly improved chemical and catalytic activities compared to the same bulk material [10].

Ongoing with these unique promising characteristics of nanoparticles, nano-sized titanium compounds are now used extensively in various consumer products, paints, pharmaceuticals, and also in wastewater treatment [11]. The cytotoxicity of several calcium titanate compounds towards different human cell lines has been also detected [4–8]. However, the cytotoxic effects of CaTiO_3_-NPs in human cancer cells have not been well studied.

Therefore, this study was undertaken to estimate the effect of CaTiO_3_ nanoparticles on the viability and genomic stability of human breast cancer cell line. Cells viability was detected using Sulforhodamine B assay and the alkaline comet assay was conducted to quantify DNA damage. Apoptosis was also estimated and cell cycle analyzed using flow cytometry. The production of intracellular reactive oxygen species was detected using 2,7-dichlorofluorescein diacetate dye, and the expression levels of the apoptotic (p53 and Bax) and anti-apoptotic (Bcl2) genes were quantified using real-time polymerase chain reaction.

## Materials and methods

### Chemicals

Calcium titanate nanoparticles (CaTiO_3_-NPs) were purchased from Sigma-Aldrich Chemical Company (Saint Louis, USA) with white appearance and product number (633801). Nano-powders of CaTiO_3_-NPs with 99% trace metals basis were suspended in deionized distilled water and ultra-sonicated to prepare the concentrations required for the experiments of this study.

### Cell lines

Human breast cancer (MCF-7) cells were purchased from Nawah Scientific Company (Mokatam Cairo Egypt) and were preserved in Dulbecco’s Modified Eagle Medium (DMEM) media provided with streptomycin (100 mg/ml), penicillin (100 units/ml) and heat-inactivated fetal bovine serum in humidified, 5% (v/v) carbon dioxide atmosphere at 37 °C.

### Characterization of CaTiO_3_-NPs

X-ray diffraction (XRD) analysis was conducted to study the properties of CaTiO_3_-NPs using a charge coupled device diffractometer (XPERT-PRO, PANalytical, Netherlands). The size distribution and Zeta potential of the CaTiO_3_-NPs were also evaluated using a Malvern Instrument Zeta sizer Nano Series (Malvern Instruments, Westborough, MA) equipped with a He–Ne laser (λ = 633 nm, max 5mW). Finally, the suspended CaTiO_3_-NPs were imaged using transmission electron microscopy (TEM) to determine the morphology and mean particle size of CaTiO_3_-NPs.

### Cells viability

The effect of CaTiO_3_-NPs on the MCF-7 cells viability was assessed using a Sulforhodamine B (SRB) assay [12]. A 100 µl of MCF-7 cell suspensions were cultured separately in 96-well plates and incubated for 24 h in complete media. After incubation, the cells were treated with five different concentrations of CaTiO_3_-NPs (0.01, 0.1, 1, 10 and 100 µg/ml) and incubated for 24 h or (0.1, 1, 10, 100 and 1000 µg/ml) and incubated for 72 h. After exposure to CaTiO_3_-NPs for 24 or 72 h, the cultured MCF-7 cells were fixed and washed with distilled water, then SRB solution (0.4% w/v) was added and the cells were incubated for 10 min at room temperature in a dark place. All plates were washed with acetic acid (1%) and left overnight to dry. The protein-bound SRB stain was then dissolved and the absorbance measured using a BMG LABTECH^®^-FLUO star Omega microplate reader (Ortenberg, Germany) at 540 nm.

### Treatment schedule

MCF-7 cells were cultured under the proper conditions and divided into control and treated cells. Control cells were treated with an equal volume of the vehicle (DMSO; final concentration, ≤ 0.1%), while treated cells were treated with the determined IC50 of CaTiO_3_-NPs. All MCF-7 cells were left for 72 h after treatment and harvested by trypsinization and centrifugation. Cells were then washed twice with ice-cold PBS and used for different molecular assays. Triplicate was done for each treatment in all conducted molecular assays.

### Genomic stability

Alkaline Comet assay was done to study the effect of CaTiO_3_-NPs on genomic stability in MCF-7 cells [13, 14]. Briefly, a mixture of cell suspensions and low melting agarose was dispersed on a clean slide covered with a layer of normal melting agarose and left to dry. Slides were then incubated in a cold lysis buffer, electrophoresed and finally immersed in neutral Tris buffer. Slides were immersed in cold absolute ethanol for permanent preparation. Immediately before examination slides are stained with ethidium bromide, photographed using epi-fluorescent microscope at magnification 200 × and fifty comet nuclei were analyzed using Comet Score TM software for each sample.

Production of intracellular ROS.

The influence of CaTiO_3_-NPs on the production level of intracellular reactive oxygen species (ROS) in MCF-7 cells was also studied based on the formation of a fluorescent dichlorofluorescein complex resulting from the reaction of a 2,7-dichlorofluorescein diacetate dye with intracellular ROS [15]. After cultured MCf-7 cells were washed with phosphate-buffered saline (PBS), 2,7-dichlorofluorescein diacetate dye was added and left for 30 min in the dark. The mixture of cells and dye was then dispersed on a clean slide and the emitted fluorescent light was examined using epi-fluorescent at 200 × magnification.

### The mRNA expression levels of p53, Bax and Bcl2 genes

The mRNA expression levels of p53, Bax and Bcl2 genes were measured using real time Polymerase chain reaction (RT-PCR) in all MCF-7 cells after 72 h of treatment. Total RNAs was extracted using the GeneJET RNA Purification Kit (Thermo scientific, USA) (Thermo scientific, USA) and then transcribed reversely into complementary DNA (cDNA) using the Revert Aid First Strand cDNA Synthesis Kit (Thermo scientific, USA). To measure mRNA expression levels, the p53, Bax and Bcl2 genes were amplified using the 7500 Fast system (Applied Biosystem 7500, Clinilab, Egypt). The primers previously designed by [16, 17] listed in Table 1 were used for RTPCR amplification and the comparative *Ct* (DD*Ct*) method was undertaken to quantify the mRNA expression levels of amplified genes. Data of RTPCR were standardized using housekeeping GAPDH gene expression and the results were expressed as mean ± S.D.

**Table 1:** Sequences of the used primers in RT-PCR

Gene	Strand	Primer’s sequences
GAPDH	Forward	5′-GAAGGTGAAGGTCGGAGTCA-3'
	Reverse	5′-GAAGATGGTGATGGGATTTC-3'
BAX	Forward	5′-CCGCCGTGGACACAGAC-3’
	Reverse	5′-CAGAAAACATGTCAGCTGCCA-3'
BCL-2	Forward	5′-TCCGATCAGGAAGGCTAGAGT-3'
	Reverse	5′-TCGGTCTCCTAAAAGCAGGC-3'
P53	Forward	5′-CAGCCAAGTCTGTGACTTGCACGTAC-3'
	Reverse	5′-CTATGTCGAAAAGTGTTTCTGTCATC-3'

### Cell cycle analysis

Analysis of MCF-7 cell cycle was conducted using flow cytometry. After 72 h of treatment MCF-7 cells were collected, washed with PBS and re-suspended in PBS with RNAase A and propidium iodide (PI). Control and treated MCF-7 cells were then incubated in dark for 20 min at 37 °C and the DNA contents were analyzed using FL2 (λex/em 535/617 nm) signal detector (ACEA Novocyte™ flow cytometer, ACEA Biosciences Inc., San Diego, CA, USA). For each sample, 12,000 events were acquired and cell cycle distribution is calculated using ACEA NovoExpress™ software (ACEA Biosciences Inc., San Diego, CA, USA).

### Induction of apoptosis

The number of apoptotic and necrotic cells were also determined using Annexin V- Fluorescein isothiocyanate (FITC) apoptosis detection kit (Abcam Inc., Cambridge Science Park Cambridge, UK) coupled with two fluorescent channels flow cytometry. After 72 h of treatment MCF-7 cells were harvested and washed with ice-cold PBS (pH 7.4). Collected cells are then incubated with Annexin V-FITC/propidium iodide (PI) solution in dark for 30 min at room temperature, injected via ACEA Novocyte™ flowcytometer (ACEA Biosciences Inc., San Diego, CA, USA) and analyzed using FL1 and FL2 signal detector for FITC and PI fluorescent signals, respectively (λex/em 488/530 nm for FITC and λex/em 535/617 nm for PI). For each sample, 12,000 events were acquired and positive FITC and/or PI cells were quantified by quadrant analysis and calculated using ACEA NovoExpress™ software (ACEA Biosciences Inc., San Diego, CA, USA).

### Statistical analysis

All results of the present study are expressed as mean ± Standard Deviation (S.D) and were analyzed using the Statistical Package for the Social Sciences (SPSS) (version 20) at the significance level p < 0.05. The *student t-test* was used to compare between the untreated and treated cancer MCF-7 cells.

## Results

### Characterization of CaTiO_3_-NPs

**Fig. 1:**
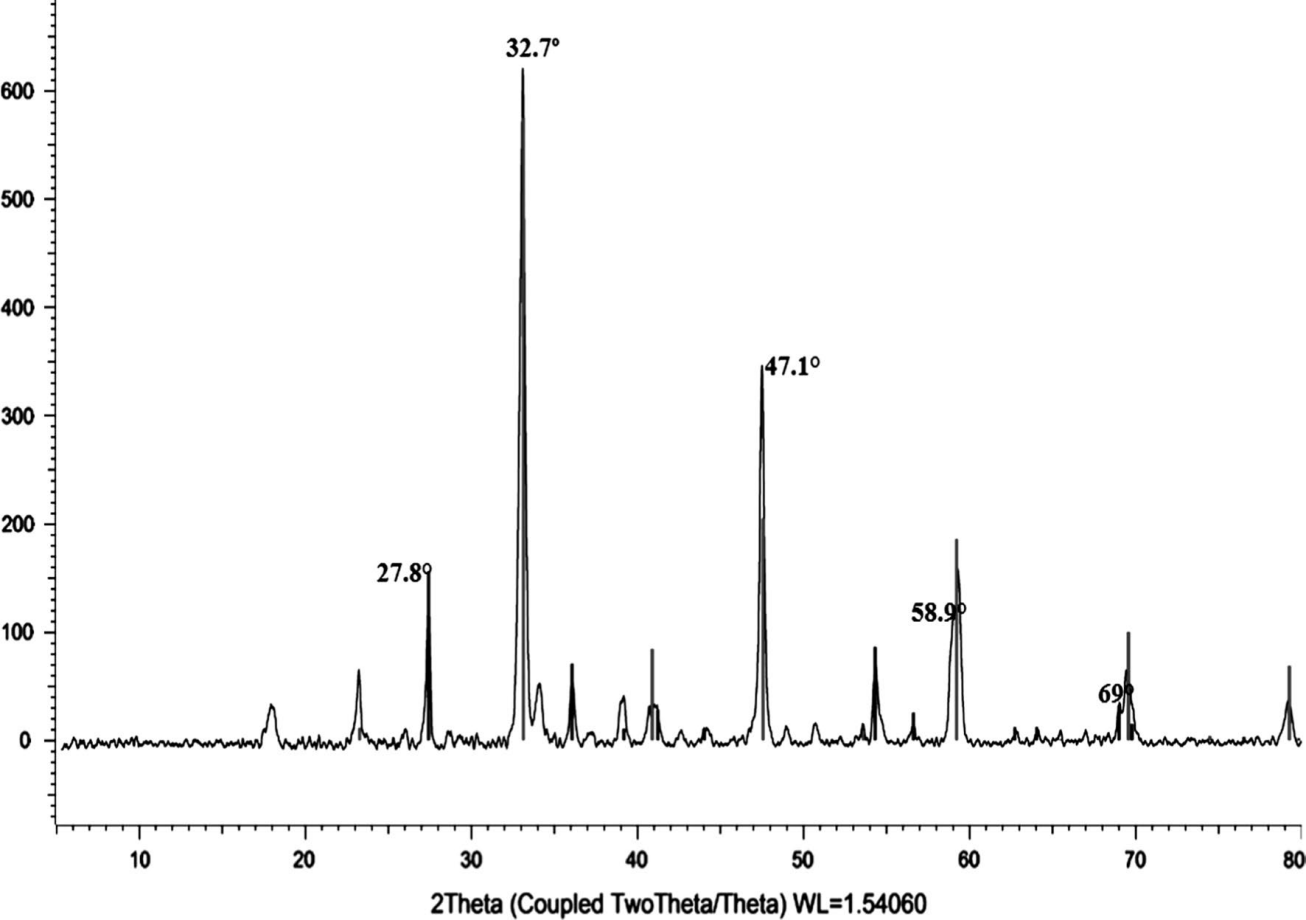
XRD pattern of calcium titanate nanoparticles showing peaks at theta angles of 32.7º, 47.1°, 58.9°, and 69°

**Fig. 2:**
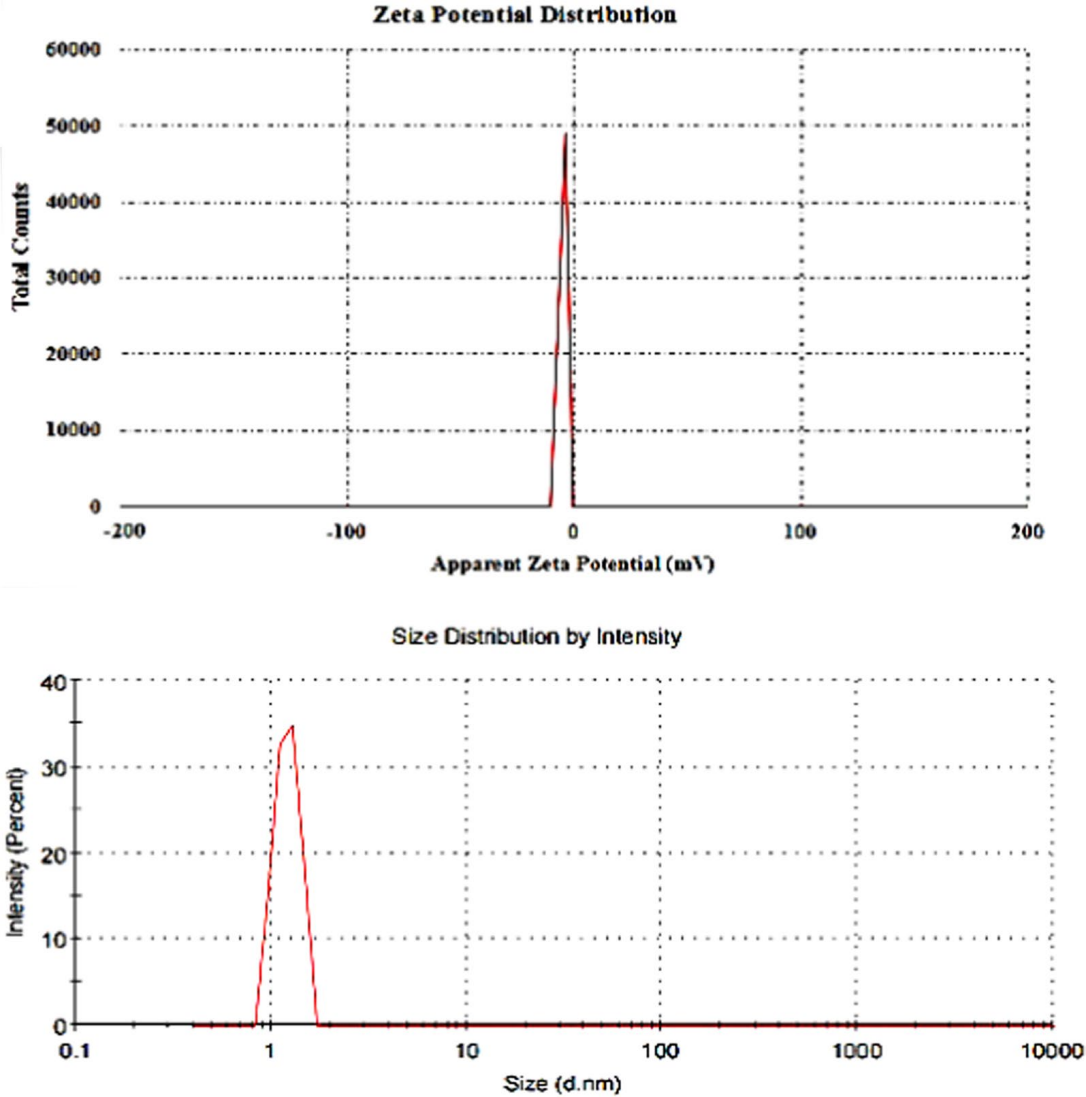
Zeta Potential and Size distribution of calcium titanate nanoparticles

**Fig. 3:**
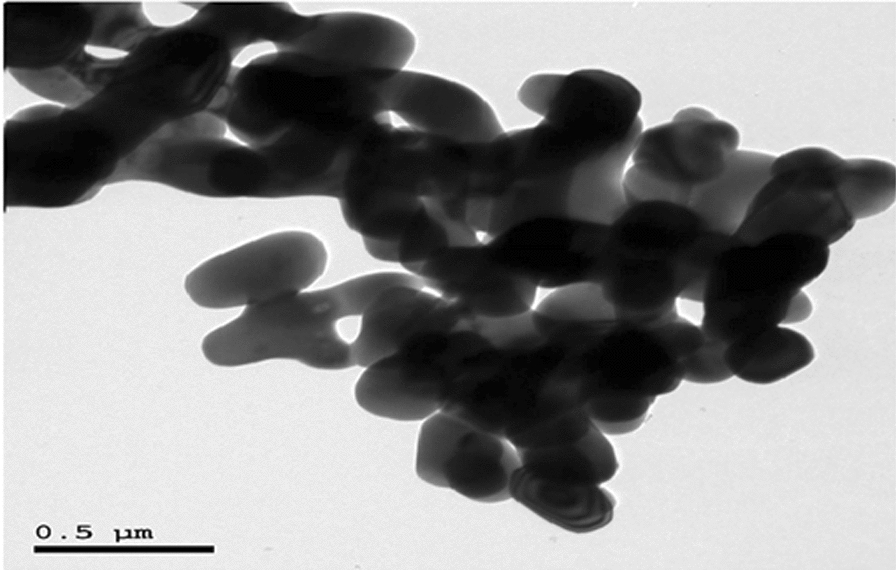
TEM image of calcium titanate nanoparticles

The results of XRD analysis confirmed the purity of the obtained CaTiO_3_-NPs and the absence of any other oxides or impurities as manifested by the appearance of CaTiO_3_ distinctive peaks at theta angles of 32.7º, 47.1°, 58.9°, and 69° (Fig. 1). Using a Zeta sizer analyzer it was found that CaTiO_3_-NPs had a mean Zeta potential value of − 3.38 mv and an average particle of 3.62 nm with a polydispersity index (PDI) value of 0.895 as seen in Fig. 2. Screening of CaTiO_3_-NPs using TEM manifested that CaTiO_3_-NPs are well dispersed and have a spherical shape (Fig. 3).

### Cells viability

**Fig. 4:**
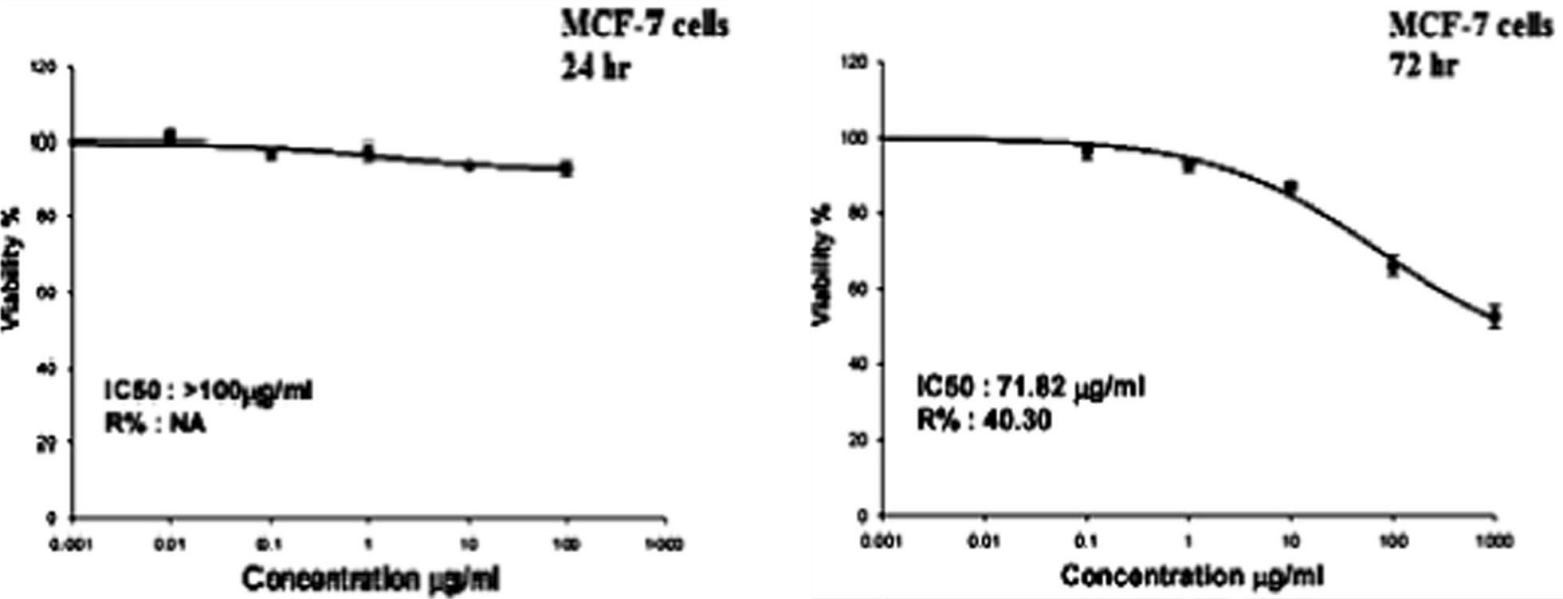
Viability of cancer MCF-7 cells after exposure to five different concentrations of calcium titanate nanoparticles (0.01, 0.1, 1, 10 and 100 µg/ml) for 24 h or (0.1, 1, 10, 100 and 1000 µg/ml) for 72 h

The results of the SRB assay showed that treatment of MCF-7 cells with CaTiO_3_-NPs concentrations 0.01, 0.1, 1, 10 and 100 µg/ml for 24 h did not affect cell viability and slight cell death was observed in cells treated with CaTiO_3_-NPs concentration greater than 10 µg/ml, and so the half maximal inhibitory concentration (IC50) of CaTiO_3_-NPs was greater than 100 µg/ml in cancer MCF-7 cells (Fig. 4). However, exposure of MCF-7 cells to CaTiO_3_-NPs five concentration 0.1, 1, 10, 100 and 1000 µg/ml for 72 h markedly decreased cells viability in a concentration dependent manner and the IC50 values for CaTiO_3_-NPs was 71.82 µg/ml in MCF-7 cells (Fig. 4).

### Genomic instability

**Fig. 5:**
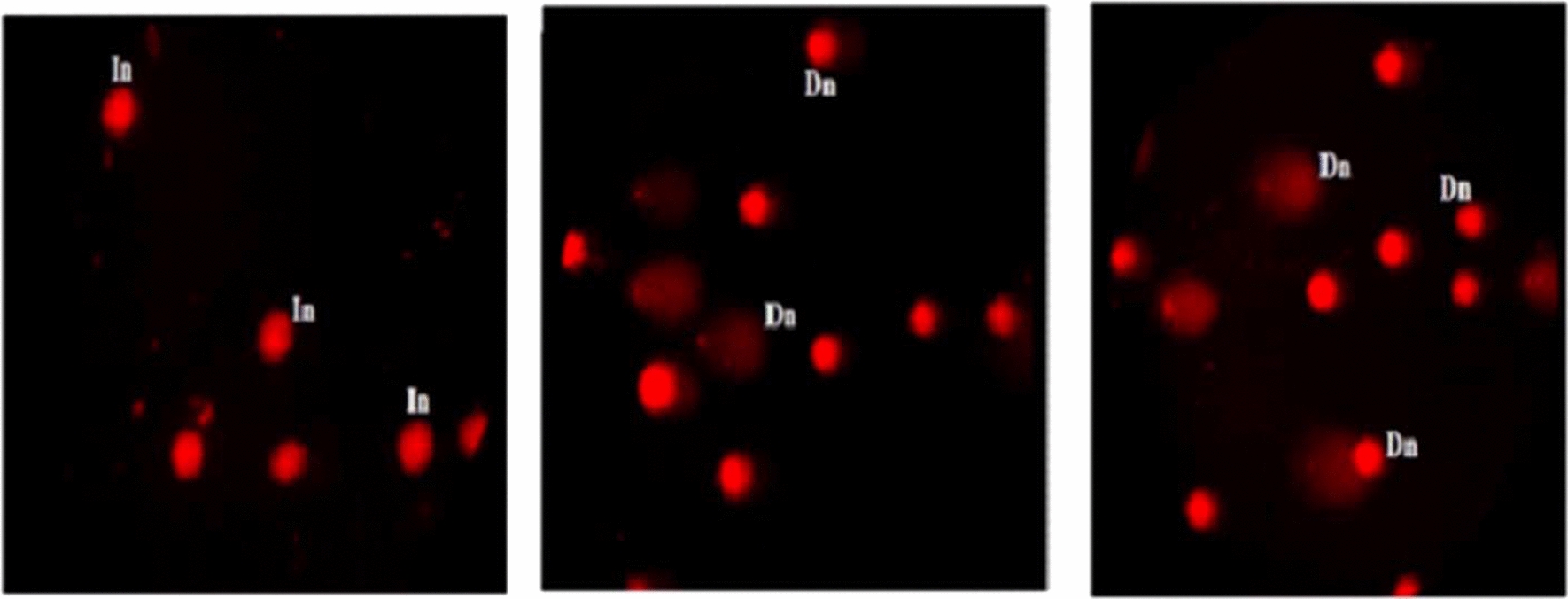
Examples for the scored Comet nuclei in MCF-7 cells showing nuclei with intact DNA (In) and other nuclei with different degree of damaged DNA damage (200 × magnification)

As displayed in Table 2 treatment of MCF-7 cancer cells with IC50 (71.82 µg/ml) of CaTiO_3_-NPs for 72 significantly increased (p < 0.05) tail length and tail moment but the % DNA in the tail was not significantly changed compared to their values in MCF-7 untreated control cells. Representative examples for the examined and scored Comet nuclei with intact and damaged DNA were shown in Fig. 5.

**Table 2:** Tail length, %DNA in tail and tail moment in the control and treated MCF-7 cells with IC50 of Calcium Titanate nanoparticles

Cell line	Treatment	Tail length	%DNA in tail	Tail moment
MCF-7	Control	5.18 ± 1.07	31.45 ± 5.4	1.93 ± 0.57
	treated	11.91 ± 0.65^a^	34.91 ± 3.09	4.89 ± 0.79^a^

Results are expressed as mean ± SD

^a^Indicates statistical significant difference from the compared control at p < 0.05 using *student t-test*

### Intracellular ROS production

**Fig. 6:**
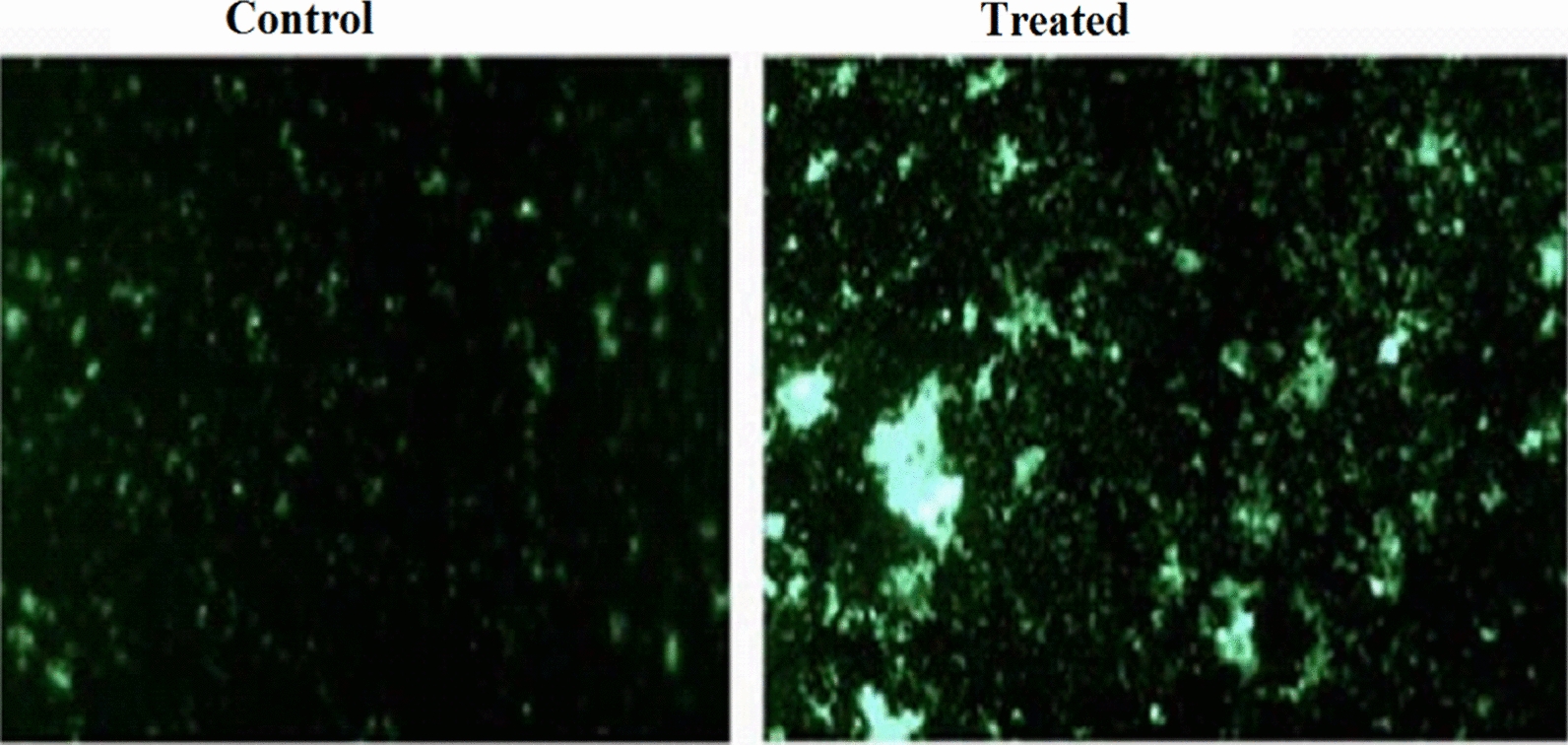
Level of ROS within the control and treated MCF-7 cells with IC50/72 of calcium titanate nanoparticles ((200 × magnification)

As shown in Fig. 6, the level of intracellular ROS production was markedly elevated in MCF-7 cancer cells treated with CaTiO_3_-NPs at a 71.82 µg/ml (IC50) concentration level compared to that of the untreated control MCF-7 cells. This high ROS generations was manifested by high increases observed in the intensities of fluorescent light emitted from MCF-7 cells stained with a 2,7-dichlorofluorescein diacetate dye compared to light emitted from untreated MCF-cells (Fig. 6).

### The mRNA expressions levels of the P53, Bax and Bcl2 genes

**Fig. 7:**
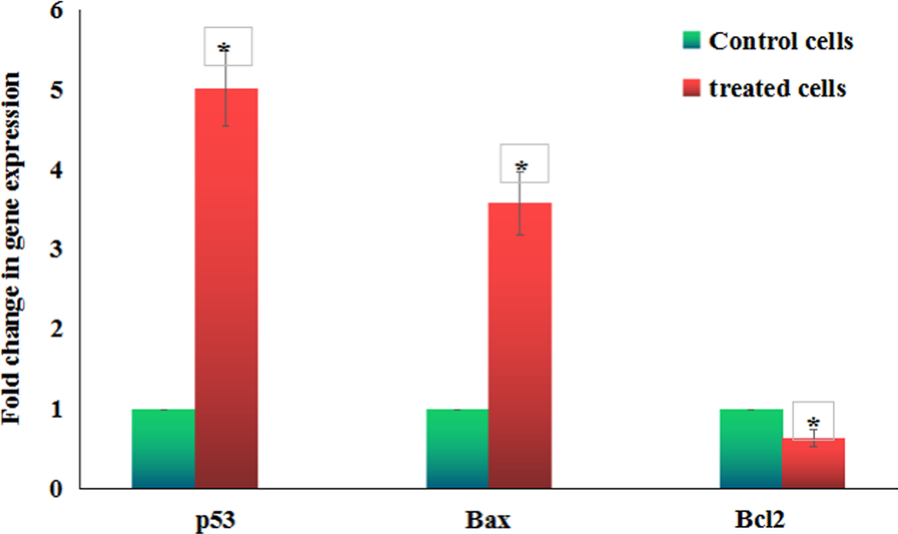
Expression levels of p53, Bax and Bcl2 genes in the control and treated MCF-7 cells with IC50/72 h of Calcium Titanate nanoparticles. Results are expressed as mean ± SD. *: Indicates statistical significant difference from the compared control at p < 0.05 using *student t-test*

Figure 7 showed that treatment with CaTiO_3_-NPs IC50 (71.82 µg/ml) for 72 h caused significant increases (p < 0.05) in the mRNA expression levels of p53 and Bax (apoptotic) genes and a significant decrease in Bcl2 (anti-apoptotic) gene expression compared to the control untreated MCF-7 expression levels.

Cell cycle distribution.

**Fig. 8:**
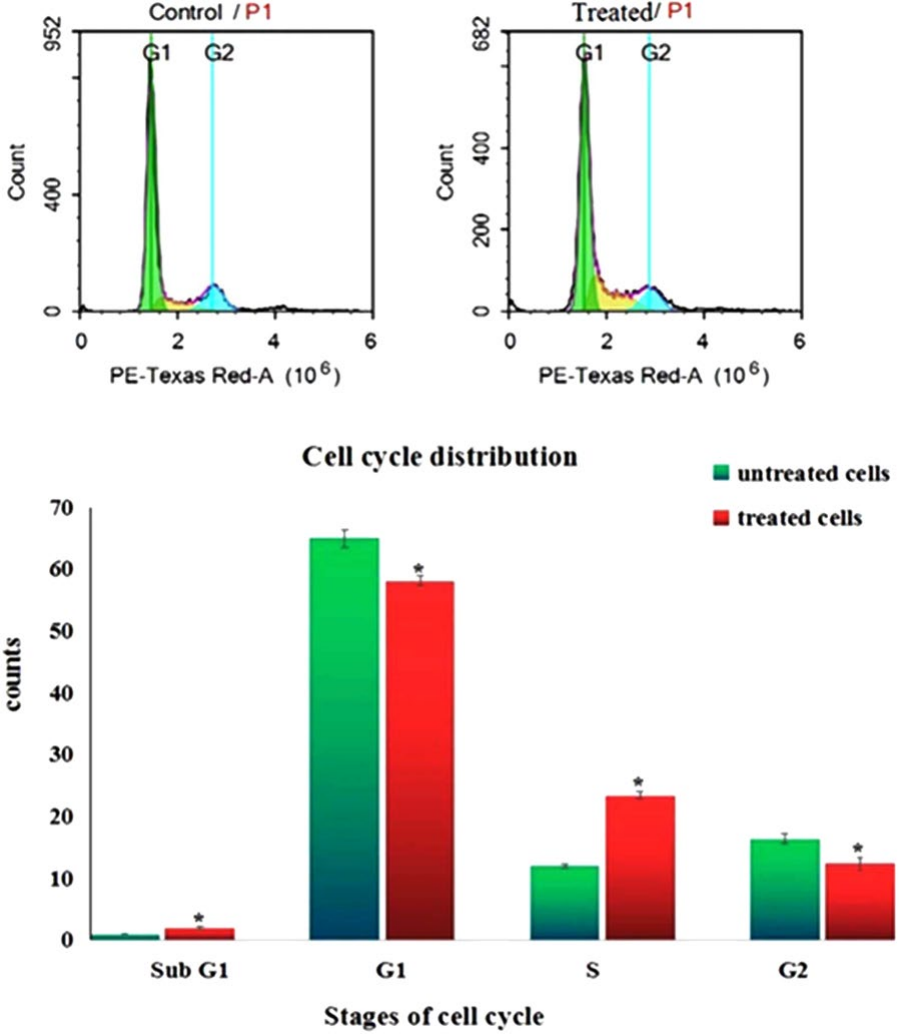
Cell cycle distribution of the control and treated MCF-7 cells with IC50/72 of calcium titanate nanoparticles

As obvious in Fig. 8 treatment of MCF-7 cells with CaTiO_3_-NPs at a concentration of 71.82 µg/ml (IC50) for 72 h caused cell cycle arrest at the G0 and S phases of cell cycle as indicated by the significant increases (p < 0.05) noticed in the number of MCF-7 cells in the SubG1 (G0) and S phases of cell cycle compared to the MCF-7 control populations in these cell cycle phases. On the contrary, the number of MCF-7 cells in the G1 and G2 phases of the cell cycle was markedly decreased after treatment with CaTiO_3_-NPs IC50 for 72 h compared to the control MCF-7 populations (Fig. 8).

### Apoptosis induction

**Fig. 9:**
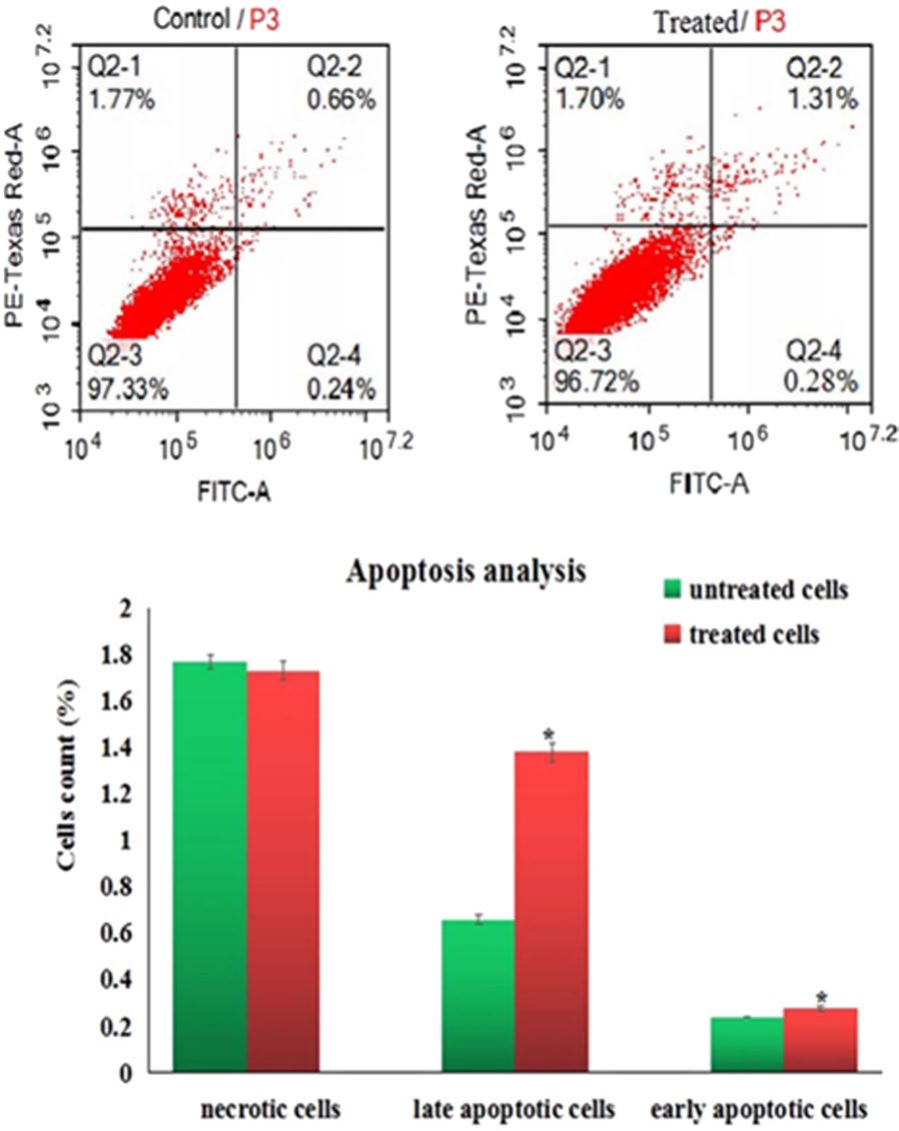
Apoptosis induction in the control and treated MCF-7 cells with IC50/72 of calcium titanate nanoparticles. Q2-1 denotes necrosis phase; Q2-2 denotes late apoptosis phase, Q2-3 denotes normal viable cells and Q2-4 denotes early apoptosis phase

As noticed in Fig. 9 exposure of MCF-7 cells to a concentration level 71.82 µg/ml (IC50) of CaTiO_3_-NPs for 72 h resulted in statistical significant elevations in the number of MCF-7 cells in the early and late apoptotic phases, while the number of necrotic MCF-7 cells was unchanged compared to the MCF-7 control cells.

## Discussion

Recent discoveries of the antioxidant and microbial activities of CaTiO_3_-NPs containing compounds together with a high capacity of CaTiO_3_ to deliver calcium ions raise interest in the use of CaTiO_3_-NPs in a wide range of medical applications [3, 18]. However, the cytotoxic and genotoxic effects of CaTiO_3_-NPs in human MCF-7 cancer cells almost have not been studied. Consequently, this study was performed to estimate CaTiO_3_-NPs induced cytotoxicity, genomic instability and apoptosis in human cancer MCF-7 cells.

Although, the size of many nanoparticles measured by DLS is actually found to be larger than the size measured by TEM, there is no fixed rule for nanoparticles because the measured size of nanoparticles depends on the concentration of nanoparticles, ions and other variables. Consequently, the lower measured size of CaTiO_3_-NPs in this study by Dynamic Laser Scattering (DLS) compared to that measured by TEM in our recent published manuscript [19] may result from using highly diluted samples in DLS and also the low Zeta potential value of − 3.38 mv makes CaTiO_3_-NPs highly aggregated in consistence with recent study [20] that showed that size of CaTiO_3_-NPs by DLS was ranged from 15–30 nm, while sized measured by TEM was larger and ranged from 10–100 nm.

The results of the SRB cytotoxicity assay demonstrated that CaTiO_3_-NPs is cytotoxic towards cancerous as manifested by the observed concentration-dependent reductions the MCF-7 cells viability after 72 h of CaTiO_3_-NPs treatment in consistent with the reported cytotoxic effects of normal-sized CaTiO_3_ in previous studies [4, 5].

The inhibition of MCF-7 cells proliferation and cytotoxic effects of CaTiO_3_-NPs demonstrated in this study can be attributed to the alterations in cell cycles distributions noticed after MCF-7 treatment with CaTiO_3_-NPs for 72 h. Our finding of the marked increases in the number of MCF-7 cells in the SubG1 (G0) and S phases of the cell cycle determined using flow cytometry revealed that CaTiO_3_-NPs caused MCF-7 cell cycle arrest at the G0/S phases and thus inhibited cells proliferation.

Reactive oxygen species (ROS) are highly reactive molecules and therefore increased ROS generations disrupt the equilibrium between oxidants and antioxidants within cells and harm cellular macromolecules: lipids, proteins, carbohydrates, and DNA [21]. Therefore, the CaTiO_3_-NPs induced toxicity described in this study may result from the marked elevations in the intracellular ROS level observed after 72 h of MCF-7 treatment with IC50 of CaTiO_3_-NPs.

Excessive ROS also attack DNA results in DNA breaks that severely damage DNA [22]. Results of Comet assay manifested DNA damage induction by CaTiO_3_-NPs through the high increases in tail length, %DNA in tail and tail moment observed after 72 h of MCF-7 exposure to IC50 of CaTiO_3_-NPs. Excessive intracellular ROS and high DNA breakages act as signals for various intracellular processes and stimulate apoptosis through upregulation of apoptotic genes and downregulation of anti-apoptotic genes expressions [23]. Consistency, apoptosis of MCF-7 cells demonstrated in this study after exposure to CaTiO_3_-NPs for 72 h by the high elevations in MCF-7 counts detected in the early and late apoptotic phases could be attributed to the above described extra-production of ROS and DNA damage induction by CaTiO_3_-NPs.

This was further confirmed by the demonstrated significant elevations in the expression levels of apoptotic p53 and Bax genes and a significant decrease in the anti-apoptotic Bcl2 gene expression after exposure of MCF-7 cells to CaTiO_3_-NPs since p53 stimulates apoptosis and regulates the expression levels of apoptotic Bax gene and the anti-apoptotic Bcl2 gene [24, 25].

The cytotoxic and genotoxic effects seen after treatment of MCF-7 cells with IC50 of CaTiO_3_-NPs for 72 h may also be explained by genomic instability and deregulation of different DNA repair pathways in highly proliferating MCF-7 cancer cells that makes MCF-7 cancer cells highly susceptible to DNA damage [26].

Similarly, treatment of normal cells with CaTiO_3_-NPs caused the time and concentration-dependent death of normal human skin fibroblast (HSF) cells. However, the genomic integrity of normal HSF cells was still normal and not disrupted after treatment with IC50/72 of CaTiO_3_-NPs for 72 h [27].

## Conclusion

Collectively from the above results, CaTiO_3_-NPs induced concentration dependent cytotoxicity in cancerous MCF-7 cells. However, exposure to CaTiO_3_-NPs IC50 inhibited MCF-7 cells proliferation through extra-generation of ROS that attack and damage genomic DNA damage stimulating apoptosis of MCF-7 cells by altering the expression levels of the p53, Bax and Bcl2 genes. More studies are thus recommended to further understand the toxic effects of CaTiO_3_-NPs.

## Data Availability

The datasets used and/or analyzed during the current study are available from the corresponding author on reasonable request.
